# Persistent Sleep Disturbance Following Pandemic Lockdowns: Longitudinal Findings From the UK COVID‐19 Symptom Study Biobank

**DOI:** 10.1111/jsr.70344

**Published:** 2026-06-08

**Authors:** Barbara Toson, Nathan J. Cheetham, Julia R. B. Brown, Carole H. Sudre, Simon Proctor, Vicky Bowyer, Nicholas R. Harvey, Desaline Joseph, COVID Symptom Study Biobank Consortium, Adam Birdseye, Claire J. Steves, Sutapa Mukherjee, Emma L. Duncan

**Affiliations:** ^1^ Adelaide Institute for Sleep Health College of Medicine and Public Health, Flinders Health and Medical Research Institute Flinders University Adelaide South Australia Australia; ^2^ Department of Twin Research and Genetic Epidemiology King's College London London UK; ^3^ James Cook University Cairns Queensland Australia; ^4^ Department of Computer Science Hawkes Institute, University College London London UK; ^5^ Unit for Lifelong Health and Ageing, Department of Population Science and Experimental Medicine University College London London UK; ^6^ Department of Biomedical Computing, School of Biomedical Engineering & Imaging Sciences King's College London London UK; ^7^ Department of Respiratory, Sleep and Ventilation Southern Adelaide Local Health Network, SA Health Bedford Park South Australia Australia; ^8^ Department of Paediatric Sleep Medicine, Evelina London Children's Health Care Guy's and St Thomas' NHS Foundation Trust London UK; ^9^ King's College London London UK; ^10^ Sleep Disorders Centre Guy's and St Thomas' NHS Foundation Trust London UK; ^11^ Department of Ageing and Health Guy's and St Thomas' NHS Foundation Trust London UK; ^12^ Department of Endocrinology Guy's and St Thomas' NHS Foundation Trust London UK

**Keywords:** COVID‐19, insomnia, post‐COVID‐19 syndrome, sleep, sleep disruption

## Abstract

Sleep disturbance and fatigue were highly prevalent during the COVID‐19 pandemic, in both infected and non‐infected individuals, though mostly assessed cross‐sectionally. To assess these symptoms after lockdown cessation, we conducted a prospective longitudinal study in a large UK cohort. Validated questionnaires assessing sleep disturbances, fatigue, depression, and anxiety were administered to COVID Symptom Study Biobank participants at two time periods—Autumn 2021 and Spring 2022 equinoxes—corresponding to lockdown and lifting of restrictions, respectively. After adjusting for differential participation using inverse probability weighting, data from 2390 participants were analysed. Generalised linear models examined SARS‐CoV‐2 infection, post‐COVID‐19 syndrome [PCS], and longitudinal changes of sleep disturbances and fatigue, adjusting for potential confounders. Despite lifting of lockdown, high levels of sleep disturbance and fatigue persisted, with no significant improvement over time, independently of baseline or new SARS‐CoV‐2 infection or PCS. *De novo* SARS‐CoV‐2 marginally worsened STOP‐Bang scores although below clinical significance. There was a trend for interaction between baseline SARS‐CoV‐2 status and Chalder Fatigue scores on follow‐up fatigue (B = 0.19, 95% CI = −0.01 to 0.39, *p* = 0.057). Receipt of booster vaccination lowered Epworth Sleepiness Scale scores (B = −0.53, 95% CI = −0.96 to −0.10, *p* = 0.016) but doubled likelihood of restless legs syndrome symptoms (OR = 2.45, 95% CI = 1.38 to 4.37, *p* = 0.002). Overall, the high rates of sleep disturbances and fatigue observed during lockdown persisted after restrictions were lifted, independent of SARS‐CoV‐2 infection. Sleep disruption reported during lockdown may endure unless actively addressed. Given the high prevalence of both sleep disturbance and fatigue, routine assessment of sleep is recommended for individuals presenting with persistent post‐pandemic fatigue, including PCS.

## 1 Introduction

The impact of the COVID‐19 pandemic continues, with ongoing SARS‐CoV‐2 infections, hospitalisations, and deaths. Many individuals report long symptom duration after COVID‐19, with fatigue the commonest symptom. The most recent UK figures (March 2023) showed 1.9 million people (2.9% of UK population) self‐reported ongoing post‐infection symptoms (‘Long COVID’) (Office of National Statistics UK 2023), though these data lack control population prevalences for meaningful comparisons (Amin‐Chowdhury and Ladhani 2021).

Widespread pandemic‐related sleep disturbance was evident from early 2020, in both infected and uninfected individuals (Jahrami et al. 2021). Sleep disturbance commonly causes fatigue; thus, a key question is whether sleep disturbance contributes to fatigue in individuals with Post‐COVID Syndrome (PCS) (World Health Organisation 2023). Our previous cross‐sectional study, in individuals with and without previous SARS‐CoV‐2 infection, found fatigue was common but not necessarily due to SARS‐CoV‐2 per se: rather, fatigue was significantly associated with validated measures of sleep disturbance, and with anxiety and depression (Proctor et al. 2025). This raised the therapeutic possibility of using proven sleep interventions for individuals with PCS, fatigue, and sleep disturbance. Relevantly, to date no effective treatment for PCS has been identified. Our findings concorded with a recent paper exploring sleep quality and fatigue in PCS, showing that many individuals with PCS experience insomnia and/or disrupted sleep patterns, exacerbating tiredness and fatigue, and hindering recovery (Jauregui‐Renaud et al. 2024). These authors also advocated a comprehensive approach to PCS that included addressing sleep issues as a critical component of managing fatigue.

Our cross‐sectional study was conducted at the 2021 autumn equinox, when the UK was experiencing ongoing regional and national lockdowns. After 27 January 2022, all UK lockdown measures ceased. We hypothesised that sleep behaviours might improve after lockdown cessation, with a return towards pre‐pandemic patterns. We therefore resurveyed our cohort at the 2022 spring equinox, assessing for longitudinal changes in validated sleep measures as lockdown measures resolved, in individuals with and without SARS‐CoV‐2 infection.

## 2 Methods

### 2.1 COVID Symptom Study Biobank

This prospective longitudinal observational cohort was volunteers from the COVID Symptom Study Biobank (CSSB) (Yorkshire and Humber NHS Research Ethics Committee Approval Ref: 20/YH/0298), itself recruited from the King's College London [KCL]‐COVID Symptom Study (KCL‐CSS)/ZOE study (Drew et al. 2020) (cohort details: Supporting Information). CSSB participants were selected from the KCL‐CSS/ZOE study according to SARS‐CoV‐2 infection status and symptom duration, emphasising recruitment of individuals with long symptom duration (Osterdahl et al. 2023).

### 2.2 Data Collection

CSSB volunteers were invited to participate in the current study (‘Sleeping Through The Pandemic’), comprising online validated sleep, physical health, and mental health questionnaires: Pittsburgh sleep quality index (PSQI) (Buysse et al. 1989), sleep condition indicator (SCI) (Espie et al. 2018), STOP‐Bang (Chung et al. 2016), Epworth Sleepiness Scale (ESS) (Johns 1991), single question screening for Restless Legs Syndrome Symptoms (RLSS) (Ferri et al. 2007), the Chalder Fatigue Scale (CFS) (Chalder et al. 1993; Cella and Chalder 2010), shortened versions of Generalised Anxiety Disorder scale (GAD‐2) (Kroenke et al. 2007) and Patient Health Questionnaire (PHQ‐2) (Gilbody et al. 2007) (questionnaire details: Table S2). Where required, formal permission to use questionnaires was sought and granted (personal correspondence, SM and ELD). The cohort was surveyed twice, before and after cessation of UK lockdowns, as close to autumn and spring equinoxes as feasible to ensure equal sunlight exposure: October 2021 (baseline) and March 2022 (follow‐up). Of note, this cohort was recruited during the pandemic; thus pre‐pandemic data were not available.

### 2.3 Inclusion Criteria

Inclusion criteria were: key demographic data (age, sex, self‐defined ethnicity, area of residence) and sufficient self‐reported SARS‐CoV‐2 test results and symptom assessments (self‐reported through the KCL‐CSS ZOE app) for disease grouping assignment (see below). Completion of both baseline and follow‐up questionnaires was required for determination of longitudinal outcomes.

### 2.4 Generation of Inverse Participation Weights

Weights representing the inverse probability of completing both baseline and follow‐up sleep questionnaires were generated from multivariable logistic regression models. Inverse probability weights (IPW) were then used to reduce potential selection bias from differential response rates, as per approaches used in previous CSSB studies (Proctor et al. 2025; Cheetham et al. 2023) (see Supporting Information). As missing data imputation techniques affect IPW robustness, these were not performed.

### 2.5 Statistical Analysis

Disease grouping, capturing SARS‐CoV‐2 infection status and symptom duration, was defined per baseline status, comprising:

- SARS‐CoV‐2 negative (irrespective of any symptom reporting)
- Short COVID‐19 (SARS‐CoV‐positive, with symptom duration < 12 weeks)
- Long COVID‐19 (SARS‐CoV‐2 positive, with symptom duration ≥ 12 weeks; i.e., WHO definition for post‐COVID‐19 syndrome; World Health Organisation 2023).

Demographic characteristics were summarised using mean and standard deviation (SD) for continuous variables, and frequencies and percentages for categorical data. Between‐group differences based on baseline SARS‐CoV‐2 results (negative/positive) were examined using independent samples *T*‐tests or chi‐square tests, as appropriate. All subsequent analyses were performed using IPW adjustments.

Sleep scores and mental health measures, both overall and stratified by SARS‐CoV‐2 infection status at baseline, were summarised at baseline and follow‐up, as were differences between these timepoints, using descriptive statistics as above. A McNemar's test was used to examine changes in RLSS between rounds.

A ‘follow‐up adjusted for baseline’ approach was used to estimate longitudinal change over time in outcomes between baseline and follow‐up (Tennant et al. 2022). Generalised linear models (GLMs) were employed to examine the influence of each measure at baseline on the same measure at follow‐up. For continuous sleep and mental health measures, we specified a GLM with a normal distribution and identity link. For categorical sleep measures (specifically, RLSS), we specified a GLM with a binomial distribution and log link. Results are presented before and after adjustment for sex, age, and body mass index [BMI] (at time of joining CSSB), and presence of chronic health problems (at time of app registration) (reduced model). Index of Multiple Deprivation (IMD) was considered as a proxy for socioeconomic status; however, inclusion in the multivariable models resulted in sparse cells and unstable estimates. IMD was therefore excluded to preserve model stability.

To investigate potential influence of baseline SARS‐CoV‐2 infection status on relationships between each sleep measure over time, the same GLMs as above were applied. The reduced model analyses included an interaction between SARS‐CoV‐2 infection status at baseline (positive vs. negative) and corresponding sleep measure at baseline. The full model analyses included anxiety and depression scores at baseline as well. The same approach was used to assess the potential effect of SARS‐CoV‐2 infection status and symptom duration at baseline.

To detect any effect from contracting SARS‐CoV‐2 between baseline and follow‐up on any difference(s) in sleep measures between the two questionnaires (Delta Δ = Follow‐up [Round 2] − Baseline [Round 1]), we applied GLMs with both reduced and full adjustments for potential confounders, considering SARS‐CoV‐2 status change between timepoints as a factor with four categories:

1. No evidence of infection (only negative tests recorded at baseline and follow‐up) [reference]
2. Initial infection only (positive at baseline only, no subsequent positive test),
3. Reinfection (positive at baseline, at least one additional positive test (≥ 90 days after initial positive test) by follow‐up), and
4. *De novo* infection (negative at baseline, with at least one positive test by follow‐up).

The ‘follow‐up adjusted for baseline’ approach was also used to determine the effect of receiving booster vaccination between baseline and follow‐up questionnaires, noting that in November 2021 (i.e., between questionnaires) the UK Joint Committee on Vaccination and Immunisation recommended booster vaccination for all adults aged ≥ 50 years.

Importantly, although the longitudinal structure allows examination of temporal relationships, this observational study cannot determine causal effect; and all results should be interpreted as associations only. Lastly, as pre‐pandemic data were not available, pre‐pandemic comparisons were not possible.

## 3 Results

### 3.1 Cohort

As shown in Figure 1, all CSSB volunteers were invited to participate; 3766 (45.11%) of 8349 completed/partially completed baseline questionnaires and subsequently 3902 (46.74%) completed follow‐up questionnaires with 2778 (73.76% of baseline participants) completing both. Of these 2778 individuals, 2390 (86.03%) had complete information on SARS‐CoV‐2 status, symptom duration, and IPWs, and comprise the current study cohort. The majority of the study cohort (1854 of 2390, 77.6%) continued to log symptoms, SARS‐CoV‐2 test results, and vaccination information regularly (at least fortnightly) between baseline and follow‐up.

**FIGURE 1:**
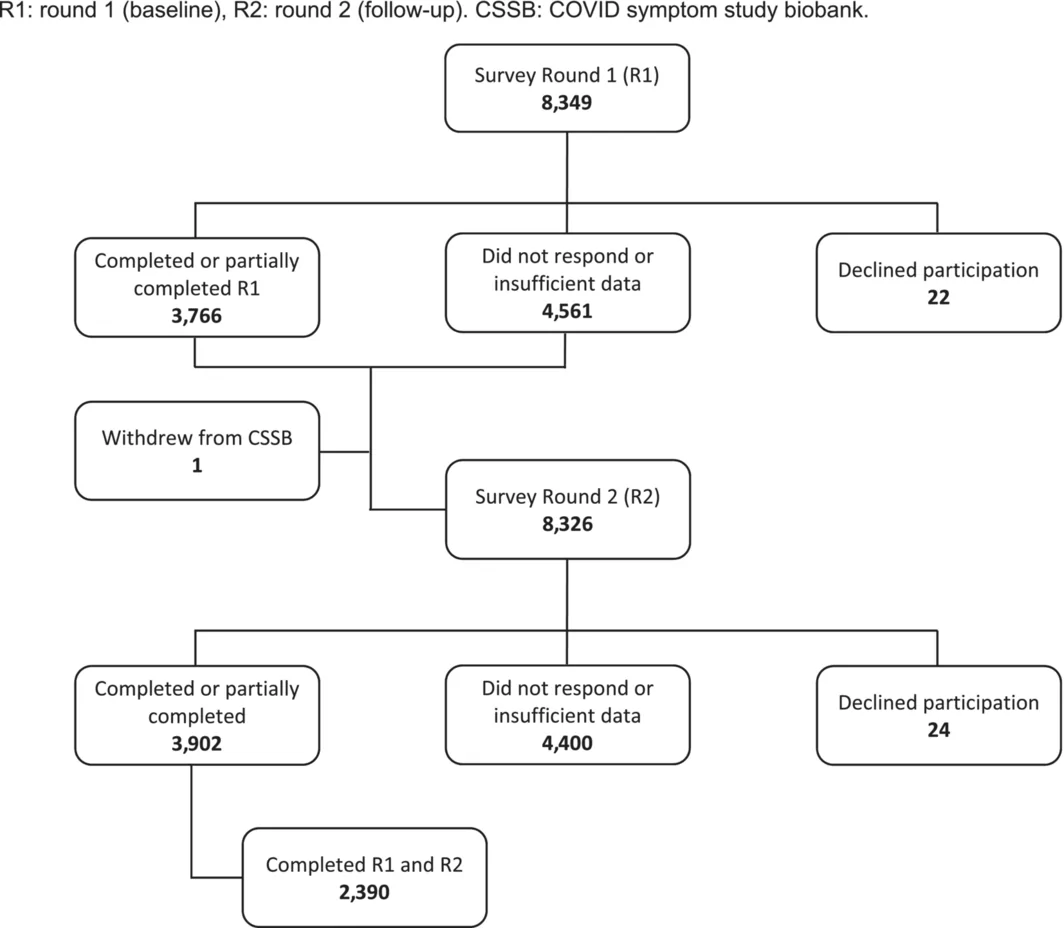
Participant recruitment to sleep study. R1: Round 1 (baseline), R2: Round 2 (follow‐up). CSSB, COVID symptom study.

Table 1 shows demographic characteristics for CSSB overall, and the current study (overall and by SARS‐CoV‐2 status). Individuals who participated in both rounds (*n* = 2390) were predominantly females (*n* = 1928, 80.7%), middle‐aged (mean age at baseline: 57 [SD 10.5] years), and from less deprived areas (IMD Quintile 5 [least deprived 20%]: *n* = 798 [33.4%]), similar to CSSB and KCL‐CSS/ZOE cohorts. Just over half of the cohort (*n* = 1474, 53.4%) had tested positive for SARS‐CoV‐2 at baseline, reflecting cohort recruitment strategy. Considering longitudinal SARS‐Co‐V status: 563 individuals (30.4%) had no evidence of infection, 760 (41.0%) had initial infection only, 353 (19.0%) had re‐infection, and 178 (9.6%) had *de novo* infection. Most participants (*n* = 2332; 97.6%) reported at least one vaccination against SARS‐CoV‐2 at baseline; the remainder (*n* = 58 [2.4%]) omitted to answer. As expected given CSSB recruitment strategy (Supporting Information), baseline symptom duration varied across groups, including long symptom duration reporting by both SARS‐CoV‐2‐positive and negative individuals.

**TABLE 1:** Demographic characteristics of participants, overall and by baseline SARS‐CoV‐2 infection status.

	CSSB cohort	Participants of both baseline and follow‐up questionnaires	SARS‐CoV‐2 status		
	Total	Total	Negative at baseline	Positive at baseline	*p‐value* ^a^
N	8349	2390	1153	1237	
Age at baseline: mean (SD)	54.2 (11.5)	58.2 (10.5)	58.4 (10.2)	58.0 (10.7)	0.310
Sex					0.150
Female	6621 (79.3%)	1928 (80.7%)	944 (81.9%)	984 (79.5%)	
Male	1717 (20.6%)	462 (19.3%)	209 (18.1%)	253 (20.5%)	
Missing	11 (0.1%)				
Baseline BMI, mean (SD)	27.1 (6.0) (*n* = 8329)	26.9 (5.9) (*n* = 2386)	26.9 (6.0) (*n* = 1151)	26.9 (5.8) (*n* = 1235)	0.815
Baseline IMD Quintile^b^					0.772
1st quintile (greatest deprivation)	525 (6.3%)	151 (6.3%)	73 (6.3%)	78 (6.3%)	
2nd quintile	1132 (13.6%)	298 (12.5%)	134 (11.6%)	164 (13.3%)	
3rd quintile	1683 (20.2%)	485 (20.3%)	233 (20.2%)	252 (20.4%)	
4th quintile	2191 (26.2%)	657 (27.5%)	318 (27.6%)	339 (27.4%)	
5th quintile (least deprivation)	2807 (33.6%)	798 (33.4%)	395 (34.3%)	403 (32.6%)	
Missing	11 (0.1%)	1 (< 1%)	0 (0.0%)	1 (0.1%)	
Baseline self‐reported ethnicity^b^					0.596
White	7989 (95.7%)	2314 (96.8%)	1119 (97.1%)	1195 (96.6%)	
Other	346 (4.1%)	73 (3.1%)	33 (2.9%)	40 (3.2%)	
Missing	14 (0.2%)	3 (0.1%)	1 (0.1%)	2 (0.2%)	
Baseline Smoker status^b^					0.129
Never	2741 (32.8%)	814 (34.1%)	364 (31.6%)	450 (36.4%)	
Former smoker	978 (11.7%)	290 (12.1%)	131 (11.4%)	159 (12.9%)	
Current smoker	156 (1.9%)	43 (1.8%)	26 (2.3%)	17 (1.4%)	
Missing	4474 (53.6%)	1243 (52.0%)	632 (54.8%)	611 (49.4%)	
Prior chronic medical condition^b^					0.657
No	6476 (77.6%)	1827 (76.4%)	886 (76.8%)	941 (76.1%)	
Yes	1873 (22.4%)	563 (23.6%)	267 (23.2%)	296 (23.9%)	
Prior mental health diagnosis^c^					0.192
No	4376 (52.4%)	1522 (63.7%)	742 (64.4%)	780 (63.1%)	
Yes	1593 (19.1%)	552 (23.1%)	287 (24.9%)	265 (21.4%)	
Missing	2380 (28.5%)	316 (13.2%)	124 (10.8%)	192 (15.5%)	
Baseline symptom duration					< 0.001
Asymptomatic	1699 (20.3%)	594 (24.9%)	417 (36.2%)	177 (14.3%)	
< 12 weeks	4751 (56.9%)	1471 (61.5%)	674 (58.5%)	797 (64.4%)	
≥ 12 weeks	773 (9.3%)	325 (13.6%)	62 (5.4%)	263 (21.3%)	
Missing (insufficient logging at baseline)	1126 (13.5%)				
Number of SARS‐CoV‐2 vaccinations at baseline					0.001
1–2	1430 (17.1%)	156 (6.5%)	56 (4.9%)	100 (8.1%)	
≥ 3	6136 (73.5%)	2176 (91.0%)	1077 (93.4%)	1099 (88.8%)	
Missing	783 (9.4%)	58 (2.4%)	20 (1.7%)	38 (3.1%)	
First documented SARS‐CoV‐2 infection					
None	2663 (31.9%)	966 (40.4%)	966 (83.8%)	0 (0.0%)	
Before 8 December 2020 (pre‐vaccination; wild‐type variant dominant)	3547 (42.5%)	851 (35.6%)	0 (0.0%)	851 (68.8%)	
First document From 8 December 2020 to 25 April 2021 (alpha‐variant dominant)	804 (9.6%)	250 (10.5%)	0 (0.0%)	250 (20.2%)	
From 26 April 2021 to 8 December 2021 (delta‐variant dominant)	411 (4.9%)	115 (4.8%)	34 (2.9%)	81 (6.5%)	
After 8 December 2021 (omicron‐variant dominant)	888 (10.6%)	208 (8.7%)	153 (13.3%)	55 (4.4%)	
Missing data	36 (0.4%)				
Booster vaccination received between rounds					< 0.001
No	2931 (35.1%)	551 (23.1%)	171 (14.8%)	380 (30.7%)	
Yes	4635 (55.5%)	1284 (53.7%)	563 (48.8%)	721 (58.3%)	
Missing (insufficient logging by baseline)	783 (9.4%)	555 (23.2%)	419 (36.3%)	136 (11.0%)	
SARS‐CoV‐2 infection between rounds (whether *de novo* or repeat infection)					< 0.001
No	1998 (23.9%)	1323 (55.4%)	563 (48.8%)	760 (61.4%)	
Yes	912 (10.9%)	531 (22.2%)	178 (15.4%)	353 (28.5%)	
Missing (insufficient logging at baseline)	5439 (65.1%)	536 (22.4%)	412 (35.7%)	124 (10.0%)	
Baseline PHQ‐2 score	1.2 (1.5) (*n* = 3166)	1.1 (1.5) (*n* = 2116)	1.1 (1.5) (*n* = 1035)	1.1 (1.4) (*n* = 1081)	0.772
Baseline GAD‐2 score	1.3 (1.6) (*n* = 3166)	1.2 (1.6) (*n* = 2116)	1.2 (1.6) (*n* = 1035)	1.3 (1.6) (*n* = 1081)	0.226

Abbreviations: BMI, body mass index; GAD‐2, generalised anxiety disorder scale 2‐item; IMD, index of multiple deprivation; PHQ‐2, patient health questionnaire 2item; R1, R1 measure.

^a^ *p*‐value are comparisons between positive and negative SARs‐CoV‐2 status, at time of baseline questionnaires. Chi‐square *p*‐value excludes missing value category from the calculation.

^b^ Collected on enrolment in KCL‐CSS/ZOE study.

^c^ Collected Jan 2021 through KCL‐CSS/ZOE study.

Systematic participation bias was evident (some regional differences; some demographic differences [less likely to participate: younger ages, higher BMI; more likely to participate: May 2021 recruitment cohort]; some health differences [more likely to participate: COVID‐positive individuals with longer (vs shorter) duration of symptoms]) (Figure S1). These biases were addressed using IPW (described above). The model generating the weighting had moderate predictive performance (AUC‐ROC = 0.76). Adjusted demographic summaries accounting for participation bias are provided in Table S3. As previously (Proctor et al. 2025), IPW‐adjusted results were used in subsequent analyses.

### 3.2 Sleep Questionnaires, Mental Health Measures, and Changes Over Time

Table 2 shows sleep questionnaire scores and mental health measures at baseline and follow‐up, for the cohort overall and by baseline SARS‐CoV‐2 status; and differences over time. At both timepoints, significant sleep disturbance was evident, with mean PSQI scores exceeding a clinically‐significant cutoff (PSQI ≥ 5). RLSS prevalence was considerably higher (approximately three to fivefold) than typical for the general population (5%–10%), at both timepoints. Other scores remained below thresholds of clinical concern (Table S2).

**TABLE 2:** Sleep questionnaire scores for individuals that participated in baseline and follow‐up questionnaires, overall and by baseline SARS‐CoV‐2 status.

	Baseline scores (Autumn equinox, 2021)		Follow‐up scores (Spring equinox, 2022)		Delta Δ (Follow‐up minus baseline)	
	Mean (SD)	Weighted counts	Mean (SD)	Weighted counts	Mean (SD)	Weighted counts
**PSQI score**						
All	7 (3.8)	2131	7.1 (3.6)	2166	0.1 (2.4)	1923
Baseline SARS‐CoV‐2 Negative	7 (3.9)	954	7.1 (3.7)	969	0.1 (2.4)	871
Baseline SARS‐CoV‐2 Positive	7 (3.7)	1177	7.1 (3.5)	1197	0.1 (2.3)	1052
**CFS score**						
All	15.5 (5.6)	2004	16.1 (6.2)	2045	0.3 (4.8)	1737
Baseline SARS‐CoV‐2 Negative	14.9 (5.6)	922	15.7 (6.2)	907	0.5 (5.4)	795
Baseline SARS‐CoV‐2 Positive	16 (5.6)	1082	16.5 (6.2)	1138	0.2 (4.3)	942
**SCI score**						
All	19.3 (8.1)	2113	19.4 (8.2)	2294	0.3 (5.3)	2032
Baseline SARS‐CoV‐2 Negative	19.1 (8.4)	952	19.2 (8.5)	1028	0.3 (5.7)	921
Baseline SARS‐CoV‐2 Positive	19.4 (7.8)	1161	19.6 (8)	1266	0.3 (5.0)	1111
**STOP‐Bang score**						
All	2 (1.5)	1731	2.1 (1.5)	1876	0 (0.8)	1478
Baseline SARS‐CoV‐2 Negative	2 (1.5)	806	2.1 (1.5)	809	0.1 (0.8)	681
Baseline SARS‐CoV‐2 Positive	2.1 (1.4)	925	2 (1.4)	1067	−0.1 (0.8)	797
**ESS score**						
All	6.4 (4.4)	2012	6.5 (4.5)	2061	0.1 (2.8)	1753
Baseline SARS‐CoV‐2 Negative	6.1 (4.2)	922	6.3 (4.3)	917	0.1 (2.7)	802
Baseline SARS‐CoV‐2 Positive	6.6 (4.5)	1089	6.8 (4.5)	1144	0.1 (2.9)	952
**PHQ‐2 score**						
All	1.2 (1.5)	2006	1.1 (1.5)	2122	0 (1.3)	1803
Baseline SARS‐CoV‐2 Negative	1.2 (1.5)	922	1.2 (1.6)	944	0 (1.4)	829
Baseline SARS‐CoV‐2 Positive	1.2 (1.4)	1084	1.1 (1.4)	1178	−0.1 (1.1)	974
**GAD‐2 score**						
All	1.3 (1.6)	2006	1.3 (1.6)	2105	0 (1.5)	1785
Baseline SARS‐CoV‐2 Negative	1.3 (1.6)	922	1.4 (1.8)	943	0.1 (1.7)	827
Baseline SARS‐CoV‐2 Positive	1.3 (1.6)	1084	1.2 (1.5)	1162	−0.1 (1.3)	958

	Weighted counts (%)	Total weighted counts	Weighted counts (%)	Total weighted counts		
**RLSS “yes”**						
All	629 (29.8%)	2109	766 (32.6%)	2348		
Baseline SARS‐CoV‐2 Negative	273 (28.7%)	950	344 (32.7%)	1052		
Baseline SARS‐CoV‐2 Positive	356 (30.7%)	1159	422 (32.6%)	1296		

*Note:* Results are adjusted for IPW. Weighted counts and % might not add up to total, due to rounding.

Abbreviations: BMI, body mass index; CFS, Chalder Fatigue Score; ESS, Epworth sleepiness scale; GAD‐2, generalised anxiety disorder scale; PHQ‐2, patient health questionnaire; PSQI, Pittsburgh sleep quality index; RLSS, restless legs syndrome symptoms; SCI, sleep condition indicator; SD, standard deviation.

For all scores, differences over time were negligible (statistically and clinically), for the cohort overall and by baseline SARS‐CoV‐2 status. While the marginal proportions of RLSS appeared similar to one another (lowest section of Table 2), considering the 2071 paired weighted counts: 208 weighted pairs reported no RLSS at baseline but had RLSS at follow‐up, while 148 weighted pairs had RLSS at baseline but not at follow‐up, indicating a significant shift in RLSS status over time, with more new cases emerging than resolving (McNemar's chi2 (1) = 10.11, *p* = 0.002).

Baseline sleep scores, fatigue scores, and mental health measures all had strongly positive associations with corresponding follow‐up results (Table S4). For the unadjusted model: *B* coefficients were 0.73–0.84 for sleep scores, 0.73 for Chalder Fatigue Scale, and 0.55 and 0.60 for GAD‐2 and PHQ‐2 scores respectively. Similar results were seen after adjustment for sex, age, BMI, and number of comorbidities per baseline data. This was particularly notable for RLSS (odds ratio [OR] = 19.2, standard error [SE] = 4.4, *p* < 0.001).

Importantly, baseline SARS‐CoV‐2 infection status did not affect the relationship between baseline sleep scores on follow‐up results (Table S5). Assessing for interaction between baseline SARS‐CoV‐2 infection status and baseline Chalder Fatigue Scale scores upon follow‐up results showed a trend towards significance both in the reduced model (*B* = 0.19, SE = 0.10, *p* = 0.057) and after the inclusion of baseline anxiety and depression measures in the model (*B* = 0.19, SE = 0.10, *p* = 0.062).

Similarly, baseline COVID‐19 symptom duration (negative, short, or long COVID‐19 status) did not significantly affect the relationship between baseline and follow‐up sleep measures (Table S6).

### 3.3 Changing SARS‐CoV‐2 Infection Status and Differences Between Baseline and Follow‐Up Scores

Table 3 shows the data according to SARS‐CoV‐2 status at baseline and follow‐up, including new SARS‐CoV‐2 infection (whether repeat or *de novo*). There was no impact on differences between rounds in sleep measures (delta Δ), with the exception of SARS‐CoV‐2 status on STOP‐Bang scores (*F*(3, 1267) = 3.69, *p* = 0.0117), which were significantly higher for individuals with *de novo* infection compared to individuals who remained negative (*B* = 0.24, 95% CI = 0.04–0.45, *p* = 0.019) or who experienced re‐infection (*B* = 0.30, 95% CI = 0.08–0.52, *p* = 0.007). However, these differences were small. Given that a clinically meaningful STOP‐Bang change is typically ≥ 3 points, the small longitudinal change in our cohort—although statistically significant—does not support meaningful clinical change.

**TABLE 3:** Changing SARS‐CoV‐2 infection status and differences between baseline and follow‐up sleep and fatigue measures.

	GLM with a normal distribution and identity link							
	Reduced models				Full models			
	B	95% CI	*p*	Weighted N	B	95% CI	*p*	Weighted *N*
**Outcome: PSQI** SARS‐CoV‐2 status				1441				1288
No infection	0.00				0.00			
Initial infection only	0.21	−0.17 to 0.58	0.274		0.12	−0.26 to 0.50	0.534	
Reinfection	−0.13	−0.57 to 0.32	0.574		−0.11	−0.58 to 0.35	0.633	
*De novo* infection	0.27	−0.44 to 0.99	0.457		0.27	−0.38 to 0.92	0.420	
**Outcome: CFS** SARS‐CoV‐2 status				1294				1274
No infection	0.00				0.00			
Initial infection only	−0.08	−1.20 to 1.04	0.890		0.31	−0.75 to 1.37	0.565	
Reinfection	0.57	−0.57 to 1.70	0.327		0.68	−0.48 to 1.84	0.253	
*De novo* infection	0.46	−0.83 to 1.76	0.483		0.09	−1.10 to 1.27	0.885	
**Outcome: SCI score** SARS‐CoV‐2 status				1523				1385
No infection	0.00				0.00			
Initial infection only	−0.02	−0.86 to 0.83	0.970		0.04	−0.78 to 0.85	0.931	
Reinfection	0.18	−0.67 to 1.02	0.684		0.26	−0.58 to 1.10	0.546	
*De novo* infection	−0.02	−1.23 to 1.19	0.977		0.02	−1.07 to 1.10	0.977	
**Outcome: STOP‐Bang** SARS‐CoV‐2 status				1100				1087
No infection	0.00				0.00			
Initial infection only	−0.09	−0.21 to 0.02	0.115		−0.08	−0.19 to 0.04	0.180	
Reinfection	−0.06	−0.20 to 0.09	0.422		−0.06	−0.21 to 0.09	0.401	
*De novo* infection	0.24	0.04–0.45	0.019		0.24	0.03–0.44	0.024	
**Outcome: ESS** SARS‐CoV‐2 status				1320				1294
No infection	0.00				0.00			
Initial infection only	0.05	−0.48 to 0.58	0.850		0.15	−0.42 to 0.72	0.611	
Reinfection	0.19	−0.30 to 0.68	0.443		0.17	−0.32 to 0.66	0.491	
*De novo* infection	0.05	−0.76 to 0.86	0.906		0.04	−0.79 to 0.87	0.924	

	GLM with a binomial distribution and log link							
	Reduced models				Full models			
	Odds ratio	95% CI	*p*	Weighted N	Odds ratio	95% CI	*p*	Weighted *N*
**Outcome: RLSS** SARS‐CoV‐2 status				1554				1410
No infection	1							
Initial infection only	0.98	0.58–1.67	0.946		0.55	0.55–1.63	0.847	
Reinfection	0.76	0.35–1.63	0.476		0.41	0.41–1.99	0.795	
*De novo* infection	1.00	0.46–2.16	0.997		0.46	0.46–2.18	0.991	

*Note:* Reduced models include baseline sleep measure, sex, age, BMI, and presence of comorbidities. Full models additionally include baseline PHQ‐2 and GAD‐2 scores. Weighted N is the sum of inverse probability weights (IPW), used to account for differential participation. Thus, small discrepancies in N across the table may be noted, consequent to weighting and rounding.

Abbreviations: BMI, body mass index; CI, confidence interval; CFS, Chalder Fatigue Score; ESS, Epworth sleepiness scale; GAD‐2, generalised anxiety disorder scale; PHQ‐2, patient health questionnaire; PSQI, Pittsburgh sleep quality index; R1, Round 1 measure; RLSS, restless legs syndrome symptoms; SCI, sleep condition indicator.

### 3.4 Receipt of Booster Vaccination and Differences Between Baseline and Follow‐Up Scores

Table 4 shows the data according to receipt of booster vaccination. Receiving booster vaccination was associated with decreased ESS (*B* = −0.53, 95% CI = −0.96 to −0.10, *p* = 0.016), but increased odds of RLSS (OR = 2.45, 95% CI 1.38–4.37, *p* = 0.002). Both results were slightly mitigated in the full model (i.e., adding in baseline measures of anxiety and depression) but remained significant. However, to contextualise: by far the strongest predictor of RLSS at follow‐up was RLSS at baseline (in the reduced model: OR = 18.43, 95% CI 11.75–28.91, *p* < 0.001, with essentially identical results in the full model), with a log‐order lower effect for booster vaccination (as above) and presence of prior comorbidities (OR = 1.97, 95% CI 0.58–1.62, *p* = 0.046) (Table S7).

**TABLE 4:** Relationship between receipt of booster vaccination and follow‐up sleep, fatigue, and mental health measures.

	GLM with a normal distribution and identity link							
	Reduced model				Full model			
	*B*	95% CI	*p*	Weighted *N*	*B*	95% CI	*p*	Weighted *N*
**PSQI**								
Booster—Yes	0.08	−0.26 to 0.43	0.627	1423	−0.03	−0.39 to 0.33	0.868	1270
**SCI**								
Booster—Yes	−0.30	−1.05 to 0.45	0.436	1506	−0.23	−0.92 to 0.46	0.511	1368
**STOP‐Bang**								
Booster—Yes	−0.07	−0.19 to 0.04	0.219	1089	−0.08	−0.20 to 0.04	0.206	1076
**ESS**								
Booster—Yes	−0.53	−0.96 to −0.10	0.016	1302	−0.50	−0.93 to −0.07	0.023	1276
**CFS**								
Booster—Yes	−0.39	−1.33 to 0.56	0.424	1276	−0.45	−1.30 to 0.40	0.297	1256
**PHQ‐2**								
Booster—Yes	0.04	−0.13 to 0.21	0.622	1329				
**GAD‐2**								
Booster—Yes	0.17	−0.03 to 0.37	0.089	1318				

	GLM with a binomial distribution and log link							
	OR	95% CI	*p*	Weighted *N*	OR	95% CI	*p*	Weighted *N*
**RLSS**								
Booster—Yes	2.45	1.38–4.37	0.002	1536	2.17	1.26–3.72	0.005	1391

*Note:* Reduced models include baseline sleep measures, sex, age, BMI, and presence of comorbidities. Full model models additionally include baseline PHQ‐2 and GAD‐2 scores. Weighted *N* is the sum of inverse probability weights (IPW), used to account for differential participation. Thus, small discrepancies in *N* across the table may be noted, consequent to weighting and rounding.

Abbreviations: BMI, body mass index; CFS, Chalder Fatigue Scale; CI, confidence intervals; CFS, Chalder Fatigue Score; ESS, Epworth sleepiness scale; GAD‐2, generalised anxiety disorder scale; PHQ‐2, Patient Health Questionnaire; PSQI, Pittsburgh sleep quality index; RLSS, restless legs syndrome symptoms; SCI, sleep condition indicator.

## 4 Discussion

Here we assessed sleep scores and mental health measures longitudinally, using validated questionnaires, in a large cohort of predominantly middle‐aged females, at two sequential equinoxes (periods with identical light exposure) but with very different social circumstances (during and after lockdown restrictions). Our cohort showed clinically significant sleep disturbance and poor mental health, with little improvement over time despite lockdown release. Neither SARS‐CoV‐2 infection status, at baseline or follow‐up, nor the presence or absence of PCS, had independent effects, suggesting that ongoing poor sleep, fatigue, and mental health of our cohort were not from SARS‐CoV‐infection per se. Our results suggest that once poor sleep was present, it was unlikely to improve without intervention.

Individuals with *de novo* SARS‐CoV‐2 infection between questionnaires had slight increases in STOP‐Bang scores. This questionnaire assesses for obstructive sleep apnoea and includes snoring and tiredness/fatigue components, which symptoms might reasonably be expected after any viral illness including COVID‐19. However, the average time between *de novo* infection and follow‐up questionnaire was 70 days.

Receiving booster vaccination slightly reduced subjective sleepiness (ESS) across all participants. Symptom improvement post‐vaccination has been reported in individuals with PCS previously (Rjoob et al. 2025). However, RLSS increased over time, and after vaccination. To contextualise our findings: large international studies showed almost doubling of RLS symptom prevalence during the pandemic (vs. pre‐pandemic rates), associated with social restrictions, insomnia, and depression (Partinen et al. 2024). We previously noted high prevalence of RLSS in our cohort at baseline (Proctor et al. 2025); the odds ratio of RLSS at follow‐up was a log‐order higher for baseline RLSS than for vaccination. RLSS are also associated with anxiety and depression (Miyaguchi et al. 2024), both of which were also common in our cohort at both time points.

Multiple studies have shown deleterious effects of the pandemic on sleep, in both infected and uninfected individuals, and across population strata internationally. Systematic reviews and meta‐analyses highlight disruption of sleep patterns globally, with widespread deterioration in sleep quality, increased insomnia symptoms, and greater use of sleep medication, especially during stringent lockdown periods (Limongi et al. 2023; Jahrami et al. 2022; Ceolin et al. 2024; Ong et al. 2021). One comprehensive review, including meta‐analysis of 250 papers with data from half a million participants and 49 countries, suggested prevalence of sleep disturbance of ~40% globally, particularly during lockdown periods, affecting 52% in individuals with COVID‐19, 46% in children and adolescents, 42% of healthcare workers, 41% in populations with special healthcare needs, 41% of university students, and 37% in general populations (noting these prevalences cannot be directly compared (Jahrami et al. 2022)).

Available data suggest that pandemic‐induced sleep disturbance generally persisted; however, most longitudinal inferences have been drawn from comparisons of cross‐sectional data: few studies have assessed sleep longitudinally and fewer still after lockdown relaxation. A large UK study assessing sleep longitudinally (8163 adults assessed at multiple time points between April 2020 and Jan 2021) and a bespoke survey (self‐report of increased sleep loss) showed marked increases in perceived sleep loss during the pandemic compared with pre‐pandemic sleep. Although prevalences did lessen over time, perceived sleep loss tracked closely with lockdown stringency; the study did not continue after complete lockdown release (Falkingham et al. 2022). A smaller study from Hong Kong assessing sleep longitudinally (339 participants at last assessment in 2021) showed significant and worsening pandemic‐related increases in insomnia symptoms and depressed mood over time; again, this study did not continue after lockdown release (Lam et al. 2021). A large early pandemic study from 2020, using data from approximately 113,000 individuals using wearable devices from 20 countries, showed poorer sleep compared with pre‐pandemic data longitudinally; and, cross‐sectionally, that stricter lockdowns were associated with poorer sleep (Ong et al. 2021); again, data were not tracked after lockdown cessation. Lastly, a longitudinal study in 216 Spanish healthcare workers showed high levels of pandemic‐induced insomnia that persisted after lockdown release; however, individuals remained vulnerable to long‐term sleep disruption from pandemic‐related stress, social isolation, and anxiety (Gil‐Almagro et al. 2024).

Thus, there is a critical data gap regarding the progression of pandemic‐induced sleep disruption, particularly post‐lockdown. Disordered sleep has a myriad of health consequences, affecting adaptive and innate immunity, inflammation, neuroendocrinology, and neuropsychiatric diseases (Medic et al. 2017; Irwin 2015). Our results concur with the broad pre‐pandemic literature: without specific intervention, sleep disturbance triggered by a precipitating factor usually persists beyond the immediate crisis; and once symptoms are habituated, spontaneous resolution is unlikely (Morin et al. 2009, 2020; Saltychev et al. 2021). Our findings reinforce and highlight the importance of monitoring for sleep disruption reported in the context of the pandemic, and, if appropriate, intervening to mitigate the known long‐term health consequences (Altena et al. 2020).

### 4.1 Strengths and Weaknesses

This study represents one of the largest longitudinal sleep cohorts to date, with strong participant retention and detailed self‐reported data on symptoms, testing, and vaccination. While we acknowledge differential participation by demographic characteristics, we employed IPW to address sampling bias due to cohort attrition. However, IPW cannot adjust for unmeasured confounding—including socioeconomic status, changes in employment, and shifts in lifestyle—cohort self‐selection, or the lack of true pre‐pandemic baseline data. Consequently, the cohort may not reflect the wider population, and generalisability is limited. Further, our cohort was predominantly white, middle‐aged, and female—consistent with CSSB and KCL‐CSS/ZOE demographics—limiting our study's generalisability to men, younger populations (including adolescents), and ethnic minorities. We also do not have employment data, including whether employment changed in the pandemic (including furlough) and/or included shift work. We acknowledge that data were entirely self‐reported and not linked to health records. Further, SARS‐CoV‐2 testing was voluntary and individualised, noting, though, that free lateral flow kits were available in the UK until 1 April 2022, covering our study period, and testing was often required by workplaces. Lastly, CSSB recruitment strategy targeted individuals with long symptom duration, restricting some quantitative approaches, although in general our large sample allowed use of parametric methods.

While sleep disturbance was common during and after lockdown, it was not associated with SARS‐CoV‐2 infection per se, or with PCS. However, without pre‐pandemic data, we cannot determine whether the high prevalence of sleep disturbance in our cohort reflects pre‐existing vulnerabilities or enduring consequences of the pandemic. Given the cohort's demographic composition—predominantly older, white women, who are reported to have higher baseline rates of insomnia and sleep disturbance compared to the general population—it is also possible that participants entered the study with higher rates of sleep‐related symptoms. Further, although we assessed participants during contrasting phases of the pandemic—from lockdown to release—the short timeframe may have been insufficient to detect sleep recovery. Nonetheless, pre‐pandemic data suggest induced sleep disorders usually persist; moreover, for many individuals lockdown was traumatic and post‐traumatic stress may have contributed to ongoing sleep disturbance (O'Regan et al. 2021). Finally, as fatigue is the commonest symptom in PCS, and poor sleep quality contributes to fatigue, our data support the potential for proven sleep therapies to help fatigue in individuals with PCS and sleep disturbance—and, soberly, that poor sleep will not improve unless this problem is actively addressed.

## 5 Conclusions

Sleep disturbance and fatigue were widespread during the pandemic lockdown and persisted after lockdown ended. Reassuringly, these were not directly related to SARS‐CoV‐2 infection. However, clinical intervention is likely to be needed to improve sleep quality in affected individuals—which may also alleviate fatigue in individuals with ongoing pandemic‐related symptoms.

## Author Contributions

**Barbara Toson:** methodology, visualization, formal analysis, writing – original draft, writing – review and editing. **Nathan J. Cheetham:** data curation, methodology, visualization, formal analysis, writing – review and editing. **Julia R. B. Brown:** data creation and curation. **Carole H. Sudre:** conceptualisation; software; data curation. **Simon Proctor:** conceptualiation; methodology. **Nicholas R. Harvey:** writing: review and editing. **Vicky Bowyer:** project administration, supervision, data curation, writing – review and editing. **Desaline Joseph:** methodology. **Adam Birdseye:** data curation; methodology. **Claire J. Steves:** CSSB conceptualisation, funding acquisition, resources, project administration, supervision. **Sutapa Mukherjee:** resources, project conceptualization, supervision, writing – review and editing. **Emma L. Duncan:** CSSB conceptualisation, funding acquisition, resources, project conceptualization, project administration, supervision, writing – original draft, writing – review and editing. All authors reviewed the final manuscript.

## Funding

This work is supported by two grants from the Denise Coates Foundation through the Chronic Disease Research Foundation (CDRF‐23/2020 The COVID Symptoms Study: The Genetics of Long COVID‐19; and CDRF‐22/2020 The COVID Symptom Study Application: Research Platform and Biobank). The KCL/ZOE COVID Symptom Study was supported by the Engineering and Physical Sciences Research Council Centre for Medical Engineering at King's College London (WT 203148/Z/16/Z), the UK Research and Innovation London Medical Imaging and Artificial Intelligence Centre for Value‐Based Healthcare, and the UK Department of Health and Social Care, including the National Institute for Health Research (NIHR)‐funded BioResource, Clinical Research Facility and comprehensive Biomedical Research Centre (BRC) award to Guy's & St Thomas' NHS Foundation Trust in partnership with King's College London and King's College Hospital NHS Foundation Trust. This research was also funded in part by the Wellcome Trust [215010/Z/18/Z]. Investigators also received support from the Chronic Disease Research Foundation (CDRF), HMT/UKRI/MRC COVID‐19 Longitudinal Health and Wellbeing—National Core Study (LHW‐NCS) (MC_PC_20030, MC_PC_20059 and NIHR COV‐LT‐0009), Medical Research Council (MRC), British Heart Foundation (BHF), Alzheimer's Society, European Union. ZOE Limited provided in‐kind support for all aspects of building, running, and supporting the app and service to users worldwide. S.O. was supported by the French government, through the 3IA Côte d'Azur Investments in the Future project managed by the National Research Agency (ANR) with the reference number ANR‐19‐P3IA‐0002. B.T. declares research income from the NHMRC (National Health and Medical Research Council. Australia). E.M. received funds from the Medical Research Council UK (Skills Development Scheme) and from the National Institute for Health Research UK (grant n.134293). TwinsUK is funded by the Wellcome Trust, Medical Research Council, Versus Arthritis, European Union Horizon 2020, Chronic Disease Research Foundation (CDRF), Wellcome Leap Dynamic Resilience Programme (co‐funded by Temasek Trust), Zoe Ltd., the National Institute for Health and Care Research (NIHR) Clinical Research Network (CRN), and Biomedical Research Centre based at Guy's and St Thomas' NHS Foundation Trust in partnership with King's College London. For the purpose of Open Access, the author has applied a CC BY public copyright licence to any Author Accepted Manuscript (AAM) version arising from this submission.

## Conflicts of Interest

Tim Spector is co‐founder and a founder shareholder of ZOE Ltd. Claire J. Steves and Sebastien Ourselin have consulted for ZOE Ltd. Barbara Toson. serves as statistical consultant to Bespoke Clinical Research. The other authors declare no conflicts of interest.

## Supporting information


**Figure S1:** Associations with completion of both sleep questionnaires.
**Table S1:**: COVID Symptom Study Biobank Consortium Named authors in current paper shown in bold.
**Table S2:**: Sleep and mental health questionnaires used in the current study.
**Table S3:**: Demographic characteristics for total cohort, and by SARS‐CoV‐2 status at baseline, after adjustment by inverse probability weighting.
**Table S4:**: Associations of baseline sleep and mental health measures on follow‐up outcomes (both unadjusted and adjusted models).
**Table S5:**: Predictors of follow‐up sleep measures, investigating interaction of baseline SARS‐CoV‐2 status and baseline sleep measures.
**Table S6:**: Predictors of follow‐up sleep scores, investigating interaction of baseline SARS‐CoV‐2 disease grouping and baseline sleep measures.
**Table S7:**: Effect of booster vaccination on restless legs syndrome.

## Data Availability

The data that support the findings of this study are not publicly available due to privacy or ethical restrictions.
