# Supplementary material for: Persistent Sleep Disturbance Following Pandemic Lockdowns: Longitudinal Findings From the UK COVID‐19 Symptom Study Biobank
Source: J Sleep Res. 2026 Jun 8;35(5):e70344. doi: 10.1111/jsr.70344 (PMC13619072; doi:10.1111/jsr.70344)
Supplement: Supplementary file 1 [file JSR-35-e70344-s001.docx]

# Persistent Sleep Disturbance Post-Lockdown: Supplementary Data

## Supplementary methods

**Cohorts: Creation and Structure**

1. **King’s College London [KCL]-COVID Symptom Study**

The King’s College London [KCL]-COVID Symptom Study (KCL-CSS)/ZOE study app was launched on 24 March 2020 (Kings College London Ethics Committee Approval LRS-19/20-18210)) and has been extensively described elsewhere (1, 2). Briefly, app users gave informed consent for use of their self-reported data for COVID-19-related research.

At registration, participants provided basic demographic information (age, sex, self-identified ethnicity per standardised Office for National Statistics categories [https://www.ons.gov.uk/methodology/classificationsandstandards/measuringequality/ethnicgroupnationalidentityandreligion], height, weight, and co-morbidities per a pre-defined broad list). Mental health data were requested in early 2021 (3).

Participants were prompted daily to log their health status, against a pre-determined list of COVID-19 symptoms. Participants could also log SARS-CoV-2 tests and results, any hospital presentation(s), and any vaccinations.

KCL/ZOE app demographic and health data are not linked to any health records.

2. **The COVID Symptom Study Biobank**

The COVID Symptom Study Biobank (CSSB) was established in September 2020 (Yorkshire & Humber NHS Research Ethics Committee Approval Ref: 20/YH/0298) and has been described extensively elsewhere (4-6). Briefly, individuals participating in the KCL-CSS/ZOE study were invited by direct email invitation to participate in CSSB, according to defined characteristics:

- UK-based adult (≥18 years)
- logging regularly (at least fortnightly), sufficient to allow calculation of symptom duration.
- at time of recruitment: known (whether positive or negative) SARS-CoV-2 status, per self-reported PCR or lateral flow antigen testing [LFAT]).

Recruitment within symptom duration and test-status groups was matched by Euclidian distance for age, sex, and body mass index [BMI]; it was not possible to match on self-reported ancestry, given the predominantly white ZOE/CSS app user base.

Initial recruitment (October-November 2020) targeted SARS-CoV-2-positive individuals with illness duration ≥4 weeks, matched to (a) test-positive individuals with asymptomatic and short illness duration, and (b) test-negative individuals with long (≥4 week) symptom duration. Subsequent recruitment (April-May 2021) further targeted these same groups but additionally recruited test-negative asymptomatic/minimally symptomatic (≤1 symptom reported around the time of testing) individuals.

Overall, 30,126 app users were invited to join CSSB, of whom 8366 enrolled and provided informed consent, including consent to KCL-CSS/ZOE app data linkage. Co-morbidity data were obtained from self-logged data at time of app registration and later questionnaires.

At time of recruitment, all individuals were pre-vaccination, and all were tested for anti-spike and anti-NC antibodies; individuals who reported test-negative status but were antibody-positive were excluded from subsequent analysis.  Mismatches between self-reported negative status and in-house positive serology result were resolved in favour of positive serology.

Further UK waves of SARS-CoV-2 infection meant infection status and symptom duration might alter; thus, group data were recalculated according to ongoing self-reported symptoms and LFAT/PCR test results (without further inhouse antibody testing), with results collected via app reporter.

CSSB demographic and health data are not linked to any health records.

## Generation of inverse participation weights

Using methods applied in previous CSSB studies (5, 6), a model that optimised prediction of questionnaire completion was found using forward sequential feature selection. The model used to predict probability of completion of both baseline and follow-up questionnaires comprised the following variables: age group, sex, self-defined ethnicity, CSSB invitation round and recruitment group, PRISMA-7 scale score (at registration with CSS app) (7), COVID-19 grouping (per SARS-CoV-2 infection status and symptom duration) at time of invitation to baseline questionnaire, hospitalisation during illness, UK region, local area deprivation, number of physical health conditions (of: asthma, lung disease, cancer, diabetes, heart disease, kidney disease – all otherwise nonspecified), and number of non-responses to CSSB studies prior to baseline sleep questionnaires. Associations between questionnaire completion and various socio-demographic and pre-existing health factors were investigated.

## Suppl. Fig. S1: Associations with completion of both sleep questionnaires.

Odds ratios with 95% confidence intervals from multivariable logistic regression models, estimating associations between socio-demographic and pre-existing health exposure variables and full completion following invitation to both baseline and follow-up sleep questionnaires. COVID-19 group variable is assigned per status at time of invitation to baseline sleep questionnaire. Results for each exposure variable presented originate from separate models that use distinct adjustment variable sets to account for potential confounding factors.

## Suppl. Table S1: COVID Symptom Study Biobank Consortium

Named authors in current paper shown in bold.

| COVID Symptom Study Bank Group | |
| --- | --- |
| **Name** | **Position (role, organisation)** |
| Michela Antonelli | Lecturer, Health Data Mining, School of Biomedical Engineering & Imaging Sciences, King’s College London, London, UK |
|  | Senior Clinical Research Manager, Department of Twin Research and Genetic Epidemiology, King’s College London, London, UK |
| **Julia R. B. Brown** | Research Assistant, Department of Twin Research and Genetic Epidemiology, King’s College London, London, UK; Medical Student, James Cook University, Australia |
| Liane S. Canas | Postdoctoral Research Associate, School of Biomedical Engineering & Imaging Sciences, King’s College London, London, UK |
| Joan Capdevila Pujol | Data Science Manager, Zoe Ltd |
| **Nathan J. Cheetham** | Senior Postdoctoral Data Scientist, Department of Twin Research and Genetic Epidemiology, King’s College London, London, UK |
| Liyuan Chen | Research Software Engineer, School of Biomedical Engineering & Imaging Sciences, King’s College London, London, UK |
| Lynn Cherkas | Genetic Epidemiologist, Department of Twin Research and Genetic Epidemiology, King’s College London, London, UK |
| Jie Deng | PhD student, School of Biomedical Engineering & Imaging Sciences, King’s College London, London, UK |
| Katie J. Doores | Department of Infectious Diseases, King’s College London, London, UK |
| **Emma L Duncan** | Professor of Clinical Endocrinology (KCL) and Honorary Consultant Physician (GSTT), Department of Twin Research and Genetic Epidemiology, King’s College London, London, UK |
| Maria Paz Garcia | Programme Manager, Department of Twin Research and Genetic Epidemiology, King’s College London, London, UK |
| Alexander Hammers | Professor of Imaging and Neuroscience (KCL), and Head of King’s College London & Guy’s and St Thomas’ PET Centre, Deputy Director of the Centre for Medical Engineering, and Honorary Consultant Physician (GSTT), School of Biomedical Engineering & Imaging Sciences, King’s College London, London, UK; King's College London & Guy's and St Thomas' PET Centre, King's College London, London, UK |
| Deborah Hart | Executive Director of TwinsUK, Department of Twin Research and Genetic Epidemiology, King’s College London, London, UK |
| **Nicholas R. Harvey** | Postdoctoral Research Associate, Department of Twin Research and Genetic Epidemiology, King’s College London, London, UK |
| Adrian Hopper | Consultant Physician, Guy’s and St Thomas’ NHS Foundation Trust, London, UK |
| Christina Hu | Head of Product, Zoe Ltd, London, UK |
| Eric Kerfoot | Postdoctoral Research Associate, School of Biomedical Engineering & Imaging Sciences, King’s College London, London, UK |
| Michael H. Malim | Senior Vice Dean for Academic Strategy & Partnerships; Co-Director of the Programme of Infection & Immunity; Professor of Infectious Diseases, Department of Infectious Diseases, King’s College London, London, UK |
| Marc Modat | Senior Lecturer, School of Biomedical Engineering & Imaging Sciences, King’s College London, London, UK |
| Erika Molteni | Senior Lecturer, School of Biomedical Engineering & Imaging Sciences, King’s College London, London, UK |
| Benjamin Murray | PhD student, School of Biomedical Engineering & Imaging Sciences, King’s College London, London, UK |
| Ayrun Nessa | Clinical Operations Manager, Department of Twin Research and Genetic Epidemiology, King’s College London, London, UK |
| Marc F Österdahl | PhD student, Department of Twin Research and Genetic Epidemiology, King’s College London, London, UK; Department of Ageing and Health, Guy’s and St Thomas’ NHS Foundation trust, London, UK |
| Sebastien Ourselin | Head of School, Biomedical Engineering & Imaging Sciences; Director, Wellcome/EPSRC Centre for Medical Engineering; Deputy Director, London Medical Imaging and Artificial Intelligence Centre for Value Based Healthcare, School of Biomedical Engineering & Imaging Sciences, King’s College London, London, UK |
| Tim D. Spector | Co-founder (ZOE); Professor of Genetic Epidemiology (KCL); and Honorary Consultant Physician (GSTT), ZOE Ltd, and Department of Twin Research and Genetic Epidemiology, King’s College London, London, UK |
| **Claire J Steves** | Professor of Ageing and Health and Clinical Director of TwinsUK (KCL); Honorary Consultant Physician (GSTT), Department of Twin Research and Genetic Epidemiology, King’s College London, London, UK; Department of Ageing and Health, Guy’s and St Thomas’ NHS Foundation trust, London, UK |
| **Carole H. Sudre** | Senior Research Fellow, MRC Unit for Lifelong Health and Ageing (UCL), School of Biomedical Engineering & Imaging Sciences, King’s College London, London, UK; MRC Unit for Lifelong Health and Ageing, Department of Population Health Sciences, University College London, London, UK Centre for Medical Image Computing, Department of Computer Science, University College London, London, UK |
| Samuel Wadge | Specialist Technical Operations Manager, Department of Twin Research and Genetic Epidemiology, King’s College London, London, UK |
| Jonathan Wolf | Co-founder and CEO, Zoe Ltd, London, UK |
| Darioush Yarand | Head of Data and IT, Department of Twin Research and Genetic Epidemiology, King’s College London, London, UK |

## Suppl. Table S2: Sleep and mental health questionnaires used in the current study.

| Questionnaire | Range | Clinical meaningful cutoff |
| --- | --- | --- |
| Pittsburgh Sleep Quality Index (8) | Total score range 0 to 21 | >5 indicates poor sleep quality and likely underlying sleep disorder |
| Chalder Fatigue Score (9, 10) | 0 to 33 using the Likert score system | ≥20 is generally considered clinically significant fatigue with ≥29 consistent with severe fatigue and possible chronic fatigue syndrome. |
| Sleep Condition Indicator (11, 12) | 0 to 32 | ≤ 16 indicates significant sleep disturbance and possible insomnia disorder. |
| STOP-Bang (13) | 0 to 8 | ≥3 is clinically significant and suggestive of moderate-high sleep apnoea risk. |
| Epworth sleepiness scale (14) | 0 to 24 | ≥10 indicates excess sleepiness. |
| Single question for Restless Legs Syndrome (15) | Binary answer: Yes or no | Yes indicates restless legs syndrome with 100% sensitivity and 97% specificity |
| Patient Health Questionnaire 2-item (16, 17) | 0 to 6 | ≥3 is consistent with depression. |
| Generalized Anxiety Disorder 2-item (18) | 0 to 6 | ≥3 is consistent with generalised anxiety. |

## Suppl. Table S3: Demographic characteristics for total cohort, and by SARS-CoV-2 status at baseline, after adjustment by inverse probability weighting.

R1: Round 1 (baseline) measure; BMI: Body Mass index; IMD: Index of Multiple Deprivation; PHQ-2: Patient Health Questionnaire; GAD-2: Generalised Anxiety Disorder scale.

**^a^** IPW inverse participation weights.

**^b^** p-values are comparisons between positive and negative SARS-CoV-2 status, at time of baseline questionnaires. P-value results are based on test of proportion for categorical variables, t-test for continuous variables, and McNemar’s test for the variable: Tested positive between R1 and R2.

^c^ collected on enrolment in KCL-CSS/ZOE study

^d^ collected Jan 2021 through KCL-CSS/ZOE study

|  | **Participants of both baseline and follow-up questionnaires** | | | |
| --- | --- | --- | --- | --- |
| **Weighed counts^a^ (%) or mean (SD)** | **Total** | **Negative at R1** | **Positive at R1** | **p-value^b^** |
| Weighed counts (%) | 2390 | 1061 (44.4%) | 1329 (55.6%) |  |
|  |  |  |  |  |
| Age at baseline: mean (SD) | 54.7 (10.7) | 55.1 (10.8) | 54.4 (10.6) | 0.327 |
|  |  |  |  |  |
| Sex |  |  |  | 0.591 |
| Female | 1920 (80.3%) | 862 (81.2%) | 1059 (79.6%) |  |
| Male | 470 (19.7%) | 199 (18.8%) | 271 (20.4%) |  |
|  |  |  |  |  |
| Baseline BMI, mean (SD) | 27.2 (6.0) | 27.1 (6.3) | 27.3 (5.7) | 0.594 |
|  |  |  |  |  |
| Baseline IMD Quintile^c^ |  |  |  | 0.350 |
| 1 (most deprivation) | 151 (6.3%) | 78 (7.4%) | 73 (5.5%) |  |
| 2 | 317 (13.3%) | 139 (13.1%) | 179 (13.4%) |  |
| 3 | 437 (18.3%) | 168 (15.8%) | 269 (20.2%) |  |
| 4 | 680 (28.4%) | 297 (28%) | 383 (28.8%) |  |
| 5 (least deprivation) | 805 (33.7%) | 380 (35.8%) | 425 (32%) |  |
| Missing | 1 (0%) | 0 (0%) | 1 (0.1%) |  |
|  |  |  |  |  |
| Baseline self-reported ethnicity^c^ |  |  |  | 0.277 |
| White | 2287 (95.7%) | 1014 (95.6%) | 1273 (95.7%) |  |
| Other | 95 (4%) | 46 (4.3%) | 49 (3.7%) |  |
| Missing | 8 (0.3%) | 1 (0%) | 7 (0.5%) |  |
|  |  |  |  |  |
| Baseline Smoker status^c^ |  |  |  | 0.549 |
| Never | 782 (32.7%) | 342 (32.3%) | 440 (33.1%) |  |
| Not currently | 297 (12.4%) | 118 (11.1%) | 179 (13.5%) |  |
| Yes | 46 (1.9%) | 25 (2.4%) | 21 (1.6%) |  |
| Missing | 1265 (52.9%) | 576 (54.3%) | 689 (51.9%) |  |
|  |  |  |  |  |
| Prior chronic medical condition (collected at Zoe enrolment) ^c^ |  |  |  | 0.932 |
| No | 1855 (77.6%) | 822 (77.5%) | 1033 (77.7%) |  |
| Yes | 535 (22.4%) | 239 (22.5%) | 296 (22.3%) |  |
|  |  |  |  |  |
| Prior mental health diagnosis^d^ (collected Jan 2021) |  |  |  | 0.002 |
| No | 1178 (49.3%) | 525 (49.5%) | 654 (49.2%) |  |
| Yes | 467 (19.6%) | 264 (24.9%) | 204 (15.3%) |  |
| Missing | 744 (31.1%) | 272 (25.6%) | 472 (35.5%) |  |
|  |  |  |  |  |
| Baseline symptom duration |  |  |  | <0.001 |
| Asymptomatic | 563 (23.6%) | 335 (14.0%) | 228 (9.6%) |  |
| <12 weeks | 1578 (66.0%) | 671 (28.1%) | 907 (38.0%) |  |
| ≥12 weeks | 249 (10.4) | 55 (2.3%) | 194 (8.1%) |  |
|  |  |  |  |  |
| Number of SARS-CoV-2 vaccinations at baseline |  |  |  | 0.323 |
| 1-2 | 242 (10.1%) | 98 (9.2%) | 144 (10.9%) |  |
| ≥3 | 1989 (83.2%) | 913 (86.1%) | 1076 (80.9%) |  |
| Missing | 159 (6.7%) | 49 (4.7%) | 110 (8.2%) |  |
|  |  |  |  |  |
| First documented SARS-CoV-2 infection |  |  |  |  |
| None | 893 (37.4%) | 893 (84.3%) |  |  |
| Before 8 December 2020 (pre-vaccination; wild-type variant dominant) | 963 (40.3%) |  | 963 (72.4%) |  |
| First document From 8 December 2020 to 25 April 2021 (alpha-variant dominant) | 241 (10.1%) |  | 241 (18.1%) |  |
| From 26 April 2021 to 8 December 2021 (delta-variant dominant) | 112 (4.7%) | 42 (4%) | 70 (5.3%) |  |
| After 8 December 2021 (omicron-variant dominant) | 181 (7.6%) | 125 (11.8%) | 56 (4.2%) |  |
| Missing data |  |  |  |  |
|  |  |  |  |  |
| Booster vaccination received between rounds |  |  |  | <0.001 |
| No | 612 (25.6%) | 183 (17.2%) | 492 (32.3%) |  |
| Yes | 1170 (48.9%) | 495 (46.6%) | 675 (50.8%) |  |
| Missing (insufficient logging by baseline timepoint) | 609 (25.5%) | 384 (36.2%) | 225 (16.9%) |  |
|  |  |  |  |  |
| SARS-CoV-2 infection between rounds |  |  |  | <0.001 |
| No | 1276 (53.4%) | 532 (50.2%) | 743 (55.9%) |  |
| Yes | 530 (22.2%) | 156 (14.7%) | 374 (28.1%) |  |
| Missing (due to insufficient logging) | 585 (24.5%) | 373 (35.1%) | 212 (16.0%) |  |
|  |  |  |  |  |
| Baseline PHQ-2 score | 1.2 (1.5) | 1.2 (1.5) | 1.2 (1.4) | 0.895 |
|  |  |  |  |  |
| Baseline GAD-2 score | 1.3 (1.6) | 1.3 (1.6) | 1.3 (1.6) | 0.607 |

## Suppl. Table S4: Associations of baseline sleep and mental health measures on follow-up outcomes (both unadjusted and adjusted models)

BMI: Body Mass index; PSQI: Pittsburgh Sleep Quality Index; CFS: Chalder Fatigue Score; SCI: Sleep Condition Indicator; ESS: Epworth sleepiness scale; PHQ-2: Patient Health Questionnaire; GAD-2: Generalised Anxiety Disorder scale; RLS: Restless Legs Syndrome Symptoms.

* Model includes age, sex, BMI and presence of comorbidities, per baseline data.

| **GLM with a normal distribution and identity link** | | | | | | |
| --- | --- | --- | --- | --- | --- | --- |
|  | **Unadjusted** | | | **Adjusted*** | | |
|  | **Coefficient** | **95% CI** | **p-value** | **Coefficient** | **95% CI** | **p-value** |
| PSQI score | 0.75 | 0.71 - 0.79 | <0.001 | 0.75 | 0.7 - 0.79 | <0.001 |
| SCI score | 0.81 | 0.77 - 0.85 | <0.001 | 0.79 | 0.76 - 0.83 | <0.001 |
| STOP-Bang score | 0.84 | 0.79 - 0.88 | <0.001 | 0.69 | 0.63 - 0.74 | <0.001 |
| ESS score | 0.82 | 0.76 - 0.88 | <0.001 | 0.81 | 0.75 - 0.87 | <0.001 |
| CFS score | 0.73 | 0.64 - 0.82 | <0.001 | 0.71 | 0.62 - 0.80 | <0.001 |
| PHQ-2 score | 0.60 | 0.53 - 0.67 | <0.001 | 0.58 | 0.51 - 0.65 | <0.001 |
| GAD-2 score | 0.55 | 0.46 - 0.64 | <0.001 | 0.53 | 0.45 - 0.61 | <0.001 |
|  | | | | | | |
| **GLM with a binomial distribution and log link** | | | | | | |
|  | **Unadjusted** | | | **Adjusted*** | | |
|  | **OR** | **95% CI** | **p-value** | **OR** | **95% CI** | **p-value** |
| RLSS | 19.2 | (12.2-30.1) | <0.001 | 19.5 | (13.0 -29.3) | <0.001 |

## Suppl. Table S5: Predictors of follow-up sleep measures, investigating interaction of baseline SARS-CoV-2 status and baseline sleep measures

BMI: Body Mass index; PHQ-2: Patient Health Questionnaire; GAD-2: Generalised Anxiety Disorder scale; PSQI: Pittsburgh Sleep Quality Index; SCI: Sleep Condition Indicator; ESS: Epworth sleepiness scale; RLSS: Restless Legs Syndrome Symptoms.

Weighted N is the sum of inverse probability weights (IPW), used to account for differential participation. Thus, small discrepancies in N across the table may be noted, consequent to weighting and rounding.

Reduced model includes age, sex, BMI and presence of comorbidities, per baseline data. Full model additionally includes PHQ-2 and GAD-2 per baseline data.

|  | **Reduced model** | | | | **Full model** | | | |
| --- | --- | --- | --- | --- | --- | --- | --- | --- |
| **GLM with a normal distribution and identity link** | **β** | **95% CI** | **p-value** | **Weighted N** | **β** | **95% CI** | **p-value** | **Weighted N** |
|  |  |  |  |  |  |  |  |  |
| PSQI |  |  |  | 1920 |  |  |  | 1806 |
| Baseline PSQI_score | 0.75 | 0.70 − 0.81 | <0.001 |  | 0.72 | 0.66 − 0.79 | <0.001 |  |
| SARS-CoV-2 negative | 0.00 |  |  |  | 0.00 |  |  |  |
| SARS-CoV-2 positive | 0.07 | -0.48 − 0.62 | 0.810 |  | 0.04 | -0.53 − 0.61 | 0.893 |  |
| SARS-CoV-2 negative # Baseline PSQI_score | 0.00 |  |  |  | 0.00 |  |  |  |
| SARS-CoV-2 positive # Baseline PSQI_score | -0.01 | -0.10 − 0.07 | 0.742 |  | -0.01 | -0.10 − 0.07 | 0.764 |  |
|  |  |  |  |  |  |  |  |  |
| SCIscore |  |  |  | 2029 |  |  |  | 1927 |
| Baseline SCI score | 0.78 | 0.71 − 0.84 | 0.000 |  | 0.76 | 0.70 − 0.82 | 0.000 |  |
| SARS-CoV-2 negative | 0.00 |  |  |  | 0.00 |  |  |  |
| SARS-CoV-2 positive | -0.50 | -2.00 − 1.01 | 0.517 |  | -0.20 | -1.70 − 1.31 | 0.798 |  |
| SARS-CoV-2 negative # Baseline SCI score | 0.00 |  |  |  | 0.00 |  |  |  |
| SARS-CoV-2 positive # Baseline SCI score | 0.03 | -0.05 − 0.11 | 0.434 |  | 0.03 | -0.05 − 0.11 | 0.497 |  |
|  |  |  |  |  |  |  |  |  |
| STOP-Bang |  |  |  | 1476 |  |  |  | 1476 |
| Baseline STOP-Bang score | 0.69 | 0.61 − 0.76 | <0.001 |  | 0.68 | 0.61 − 0.75 | <0.001 |  |
| SARS-CoV-2 negative | 0.00 |  |  |  | 0.00 |  |  |  |
| SARS-CoV-2 positive | -0.08 | -0.24 − 0.08 | 0.325 |  | -0.08 | -0.24 − 0.08 | 0.326 |  |
| SARS-CoV-2 negative # Baseline STOP-Bang score | 0.00 |  |  |  | 0.00 |  |  |  |
| SARS-CoV-2 positive # Baseline STOP-Bang score | 0.00 | -0.09 − 0.09 | 0.991 |  | 0.00 | -0.09 − 0.09 | 0.981 |  |
|  |  |  |  |  |  |  |  |  |
| ESS |  |  |  | 1750 |  |  |  | 1745 |
| Baseline ESS score | 0.81 | 0.73 − 0.89 | <0.001 |  | 0.81 | 0.73 − 0.89 | <0.001 |  |
| SARS-CoV-2 negative | 0.00 |  |  |  | 0.00 |  |  |  |
| SARS-CoV-2 positive | 0.01 | -0.72 − 0.74 | 0.977 |  | 0.05 | -0.68 − 0.77 | 0.902 |  |
| SARS-CoV-2 negative # Baseline ESS score | 0.00 |  |  |  | 0.00 |  |  |  |
| SARS-CoV-2 positive # Baseline ESS score | 0.00 | -0.11 − 0.11 | 0.995 |  | -0.01 | -0.12 − 0.10 | 0.884 |  |
|  |  |  |  |  |  |  |  |  |
| CFS |  |  |  | 1734 |  |  |  | 1734 |
| Baseline CFS score | 0.60 | 0.41 − 0.79 | <0.001 |  | 0.57 | 0.35 − 0.79 | <0.001 |  |
| SARS-CoV-2 negative | 0.00 |  |  |  | 0.00 |  |  |  |
| SARS-CoV-2 positive | -3.03 | -5.65 − -0.40 | 0.024 |  | -2.88 | -5.46 − 0.31 | 0.028 |  |
| SARS-CoV-2 negative # Baseline CFS score | 0.00 |  |  |  | 0.00 |  |  |  |
| SARS-CoV-2 positive # Baseline CFS score | 0.19 | -0.01 − 0.39 | 0.057 |  | 0.19 | -0.01 − 0.38 | 0.062 |  |
|  |  |  |  |  |  |  |  |  |
|  |  |  |  |  |  |  |  |  |
|  |  |  |  |  |  |  |  |  |
| **GLM with a binomial distribution and log link** | **Odds Ratio** | **95% CI** | **p-value** |  | **Odds Ratio** | **95% CI** | **p-value** |  |
|  |  |  |  |  |  |  |  |  |
| RLSS |  |  |  |  |  |  |  |  |
| Baseline RLSS no (ref) | 1 |  |  |  |  |  |  |  |
| Baseline RLSS yes | 17.89 | 9.64 − 33.20 | <0.001 |  | 17.31 | 9.45 − 31.71 | <0.001 |  |
| SARS-CoV-2 negative | 1 |  |  |  | 1 |  |  |  |
| SARS-CoV-2 positive | 0.89 | 0.46 − 1.7 | 0.719 |  | 0.86 | 0.44 − 1.66 | 0.645 |  |
| SARS-CoV-2 negative # Baseline RLSS no (ref) | 1 |  |  |  |  |  |  |  |
| SARS-CoV-2 positive # Baseline RLSS yes | 1.18 | 0.49 − 2.85 | 0.719 |  | 1.43 | 0.61 − 3.36 | 0.413 |  |

## Suppl. Table S6: Predictors of follow-up sleep scores, investigating interaction of baseline SARS-CoV-2 disease grouping and baseline sleep measures

BMI: Body Mass index; IMD: Index of Multiple Deprivation; PHQ-2: Patient Health Questionnaire; GAD-2: Generalised Anxiety Disorder scale; PSQI: Pittsburgh Sleep Quality Index; SCI: Sleep Condition Indicator; ESS: Epworth sleepiness scale; RLSS: Restless Legs Syndrome Symptoms, CFS: Chalder Fatigue Scale.

R1 COVID-19 disease grouping distinguishes between SARS-CoV-2 negative (irrespective of symptom duration), Short COVID-19 (SARS-CoV-positive with symptom duration <12 weeks), and Long COVID-19 (SARS-CoV-2 positive, with symptom duration ≥ 12 weeks).

Reduced model includes age, sex, BMI and presence of comorbidities, per baseline data. Full model additionally includes PHQ-2 and GAD-2 per baseline data.

|  | **Reduced model** | | | **Full model** | | |
| --- | --- | --- | --- | --- | --- | --- |
| **GLM with a normal distribution and identity link** | **B** | **95% CI** | **p-value** | **B** | **95% CI** | **p-value** |
|  |  |  |  |  |  |  |
| PSQI score |  |  |  |  |  |  |
| Baseline PSQI score | 0.75 | 0.70 − 0.81 | <0.001 | 0.72 | 0.66 − 0.79 | <0.001 |
| SARS-CoV-2 neg any symptoms | 0.00 |  |  | 0.00 |  |  |
| Short COVID-19 | 0.10 | -0.49 − 0.69 | 0.743 | 0.08 | -0.54 − 0.70 | 0.796 |
| Long COVID-19 | -0.07 | -0.84 − 0.69 | 0.854 | -0.17 | -0.95 − 0.61 | 0.667 |
| SARS-CoV-2 neg any symptoms # Baseline PSQI score | 0.00 |  |  | 0.00 |  |  |
| Short COVID-19 # Baseline PSQI score | -0.02 | -0.11 − 0.08 | 0.681 | -0.02 | -0.12 − 0.08 | 0.686 |
| Long COVID-19 # Baseline PSQI score | 0.01 | -0.09 − 0.11 | 0.874 | 0.02 | -0.08 − 0.12 | 0.753 |
|  |  |  |  |  |  |  |
| SCI score |  |  |  |  |  |  |
| Baseline SCI score | 0.78 | 0.71 − 0.84 | <0.001 | 0.76 | 0.70 − 0.82 | <0.001 |
| SARS-CoV-2 neg any symptoms | 0.00 |  |  | 0.00 |  |  |
| Short COVID-19 | -0.36 | -1.99 − 1.27 | 0.665 | 0.01 | -1.60 − 1.63 | 0.986 |
| Long COVID-19 | -0.85 | -3.02 − 1.32 | 0.442 | -0.71 | -2.91 − 1.49 | 0.529 |
| SARS-CoV-2 neg any symptoms # Baseline SCI score | 0.00 |  |  | 0.00 |  |  |
| Short COVID-19 # Baseline SCI score | 0.03 | -0.05 − 0.11 | 0.495 | 0.02 | -0.06 − 0.10 | 0.585 |
| Long COVID-19 # Baseline SCI score | 0.03 | -0.09 − 0.15 | 0.656 | 0.02 | -0.10 − 0.14 | 0.732 |
|  |  |  |  |  |  |  |
| STOP-Bang |  |  |  |  |  |  |
| Baseline STOP-Bang score | 0.69 | 0.61 − 0.76 | <0.001 | 0.68 | 0.61 − 0.75 | <0.001 |
| SARS-CoV-2 neg any symptoms | 0.00 |  |  | 0.00 |  |  |
| Short COVID-19 | -0.07 | -0.25 − 0.10 | 0.404 | -0.07 | -0.24 − 0.10 | 0.417 |
| Long COVID-19 | -0.12 | -0.39 − 0.14 | 0.368 | -0.13 | -0.40 − 0.14 | 0.336 |
| SARS-CoV-2 neg any symptoms # Baseline STOP-Bang score | 0.00 |  |  | 0.00 |  |  |
| Short COVID-19 # Baseline STOP-Bang score | 0.00 | -0.10 − 0.10 | 0.973 | 0.00 | -0.10 − 0.10 | 0.956 |
| Long COVID-19 # Baseline STOP-Bang score | 0.01 | -0.14 − 0.16 | 0.934 | 0.01 | -0.14 − 0.16 | 0.917 |
|  |  |  |  |  |  |  |
| ESS score |  |  |  |  |  |  |
| Baseline ESS score | 0.81 | 0.73 − 0.89 | <0.001 | 0.81 | 0.73 − 0.89 | <0.001 |
| SARS-CoV-2 neg any symptoms | 0.00 |  |  | 0.00 |  |  |
| Short COVID-19 | 0.03 | -0.76 − 0.82 | 0.943 | 0.06 | -0.73 − 0.84 | 0.883 |
| Long COVID-19 | -0.07 | -0.96 − 0.82 | 0.877 | 0.00 | -0.90 − 0.89 | 0.992 |
| SARS-CoV-2 neg any symptoms # Baseline ESS score | 0.00 |  |  | 0.00 |  |  |
| Short COVID-19 # Baseline ESS score | 0.00 | -0.13 − 0.12 | 0.961 | -0.01 | -0.14 − 0.11 | 0.862 |
| Long COVID-19 # Baseline ESS score | 0.01 | -0.12 − 0.14 | 0.877 | 0.00 | -0.13 − 0.14 | 0.984 |
|  |  |  |  |  |  |  |
| CFS score |  |  |  |  |  |  |
| Baseline CFS score | 0.60 | 0.41 − 0.79 | <0.001 | 0.57 | 0.35 − 0.79 | <0.001 |
| SARS-CoV-2 neg any symptoms | 0.00 |  |  | 0.00 |  |  |
| Short COVID-19 | -3.18 | -5.88 − -0.48 | 0.021 | -3.00 | -5.66 − -0.35 | 0.027 |
| Long COVID-19 | -0.99 | -4.30 − 2.32 | 0.558 | -1.02 | -4.32 − 2.28 | 0.544 |
| SARS-CoV-2 neg any symptoms # Baseline Chalder score | 0.00 |  |  | 0.00 |  |  |
| Short COVID-19 # Baseline Chalder score | 0.20 | -0.01 − 0.40 | 0.064 | 0.19 | -0.02 − 0.39 | 0.074 |
| Long COVID-19 # Baseline Chalder score | 0.12 | -0.10 − 0.34 | 0.270 | 0.13 | -0.10 − 0.35 | 0.264 |
|  |  |  |  |  |  |  |
| **GLM with a binomial distribution and log link** | **Odds Ratio** | **95% CI** | **p-value** | **Odds Ratio** | **95% CI** | **p-value** |
|  |  |  |  |  |  |  |
| RLSS |  |  |  |  |  |  |
| Baseline RLSS no | 1 |  |  | 1 |  |  |
| Baseline RLSS yes | 17.93 | 9.67 − 33.23 | <0.001 | 17.38 | 9.5 3 − 1.77 | <0.001 |
| SARS-CoV-2 negative | 1 |  |  | 1 |  |  |
| SARS-CoV-2 positive short duration | 0.88 | 0.44 − 1.75 | 0.712 | 0.85 | 0.41 − 1.72 | 0.644 |
| SARS-CoV-2 positive long duration | 0.95 | 0.45 − 2.00 | 0.892 | 0.92 | 0.44 − 1.93 | 0.835 |
| SARS-CoV-2 negative # Baseline RLSS no | 1 |  |  | 1 |  |  |
| SARS-CoV-2 positive short duration # Baseline RLSS yes | 1.02 | 0.4 2 −0.63 | 0.962 | 1.24 | 0.5 3.07 | 0.639 |
| SARS-CoV-2 positive long duration # Baseline RLSS yes | 2.12 | 0.76 − 5.91 | 0.152 | 2.45 | 0.86 6.98 | 0.093 |
|  |  |  |  |  |  |  |

## Suppl. Table S7: Effect of booster vaccination on restless legs syndrome

BMI: Body Mass index; CI: confidence interval. RLSS: Restless Legs Syndrome Symptoms; PHQ-2: Patient Health Questionnaire; GAD-2: Generalised Anxiety Disorder scale.

Weighted N is the sum of inverse probability weights (IPW), used to account for differential participation. Thus, small discrepancies in N across the table may be noted, consequent to weighting and rounding.

Reduced model includes baseline sleep measures, and age, sex, BMI, and presence of comorbidities, per baseline data. Full model additionally includes PHQ-2 and GAD-2 per baseline data.

|  | **GLM with a binomial distribution and log link** | | | | | |
| --- | --- | --- | --- | --- | --- | --- |
|  | **Reduced model** | | | **Full model** | | |
|  | **Weighted N= 1536** | | | **Weighted N= 1391** | | |
|  | **OR** | **95% CI** | **p-value** | **OR** | **95% CI** | **p-value** |
|  |  |  |  |  |  |  |
| Booster - Yes | 2.45 | 1.38 − 4.37 | 0.002 | 2.17 | 1.26 − 3.73 | 0.005 |
| Male | 0.97 | 0.58 − 1.62 | 0.912 | 0.98 | 0.58 − 1.64 | 0.934 |
| Age | 0.99 | 0.97 − 1.02 | 0.677 | 1.00 | 0.97 − 1.02 | 0.806 |
| BMI | 1.01 | 0.97 − 1.04 | 0.763 | 1.00 | 0.97 − 1.03 | 0.992 |
| Baseline RLSS yes | 18.43 | 11.75 − 28.91 | <0.001 | 18.42 | 11.37 − 29.86 | 0.000 |
| Prior chronic medical condition - yes | 1.97 | 1.01 − 3.85 | 0.046 | 1.98 | 1.03 − 3.81 | 0.041 |
| PHQ-2 score |  |  |  | 0.92 | 0.73 − 1.16 | 0.487 |
| GAD-2 score |  |  |  | 1.25 | 1.00 − 1.57 | 0.047 |

**Supplementary Section References**

1.	Menni C, Sudre CH, Steves CJ, Ourselin S, Spector TD. Quantifying additional COVID-19 symptoms will save lives. Lancet. 2020;395(10241):e107-e8.

2.	Sudre CH, Murray B, Varsavsky T, Graham MS, Penfold RS, Bowyer RC, et al. Attributes and predictors of long COVID. Nat Med. 2021;27(4):626-31.

3.	Klaser K, Thompson EJ, Nguyen LH, Sudre CH, Antonelli M, Murray B, et al. Anxiety and depression symptoms after COVID-19 infection: results from the COVID Symptom Study app. J Neurol Neurosurg Psychiatry. 2021;92(12):1254-8.

4.	Osterdahl MF, Whiston R, Sudre CH, Asnicar F, Cheetham NJ, Blanco Miguez A, et al. Metabolomic and gut microbiome profiles across the spectrum of community-based COVID and non-COVID disease. Sci Rep. 2023;13(1):10407.

5.	Cheetham NJ, Penfold R, Giunchiglia V, Bowyer V, Sudre CH, Canas LS, et al. The effects of COVID-19 on cognitive performance in a community-based cohort: a COVID symptom study biobank prospective cohort study. EClinicalMedicine. 2023;62:102086.

6.	Proctor S, Cheetham NJ, Brown JRB, Bowyer V, Toson B, Harvey NR, et al. Pandemic-disordered sleep: longer illness and more fatigue but little SARS-CoV-2 effect. ERJ Open Res. 2025;11(4).

7.	Raiche M, Hebert R, Dubois MF. PRISMA-7: a case-finding tool to identify older adults with moderate to severe disabilities. Arch Gerontol Geriatr. 2008;47(1):9-18.

8.	Buysse DJ, Reynolds CF, 3rd, Monk TH, Berman SR, Kupfer DJ. The Pittsburgh Sleep Quality Index: a new instrument for psychiatric practice and research. Psychiatry Res. 1989;28(2):193-213.

9.	Chalder T, Berelowitz G, Pawlikowska T, Watts L, Wessely S, Wright D, et al. Development of a fatigue scale. J Psychosom Res. 1993;37(2):147-53.

10.	Cella M, Chalder T. Measuring fatigue in clinical and community settings. J Psychosom Res. 2010;69(1):17-22.

11.	Espie CA, Kyle SD, Hames P, Gardani M, Fleming L, Cape J. The Sleep Condition Indicator: a clinical screening tool to evaluate insomnia disorder. BMJ Open. 2014;4(3):e004183.

12.	Espie CA, Farias Machado P, Carl JR, Kyle SD, Cape J, Siriwardena AN, et al. The Sleep Condition Indicator: reference values derived from a sample of 200 000 adults. J Sleep Res. 2018;27(3):e12643.

13.	Chung F, Abdullah HR, Liao P. STOP-Bang Questionnaire: A Practical Approach to Screen for Obstructive Sleep Apnea. Chest. 2016;149(3):631-8.

14.	Johns MW. A new method for measuring daytime sleepiness: the Epworth sleepiness scale. Sleep. 1991;14(6):540-5.

15.	Ferri R, Lanuzza B, Cosentino FI, Iero I, Tripodi M, Spada RS, et al. A single question for the rapid screening of restless legs syndrome in the neurological clinical practice. Eur J Neurol. 2007;14(9):1016-21.

16.	Kroenke K, Spitzer RL, Williams JB. The PHQ-9: validity of a brief depression severity measure. J Gen Intern Med. 2001;16(9):606-13.

17.	Kroenke K, Spitzer RL, Williams JB. The Patient Health Questionnaire-2: validity of a two-item depression screener. Med Care. 2003;41(11):1284-92.

18.	Kroenke K, Spitzer RL, Williams JB, Monahan PO, Lowe B. Anxiety disorders in primary care: prevalence, impairment, comorbidity, and detection. Ann Intern Med. 2007;146(5):317-25.
